# The Photoperiod-Insensitive Allele *Ppd-D1a* Promotes Earlier Flowering in *Rht12* Dwarf Plants of Bread Wheat

**DOI:** 10.3389/fpls.2018.01312

**Published:** 2018-10-22

**Authors:** Liang Chen, Yingying Du, Qiumei Lu, Hua Chen, Ruishuang Meng, Chunge Cui, Shan Lu, Yang Yang, Yongmao Chai, Juan Li, Lulu Liu, Xiangning Qi, Hang Li, Kohei Mishina, Fei Yu, Yin-Gang Hu

**Affiliations:** ^1^State Key Laboratory of Crop Stress Biology in Arid Areas and College of Agronomy, Northwest A&F University, Xianyang, China; ^2^Department of Agricultural, Food and Nutritional Science, University of Alberta, Edmonton, AB, Canada; ^3^National Institute of Agrobiological Sciences, Tsukuba, Japan; ^4^College of Life Sciences, Northwest A&F University, Xianyang, China; ^5^Institute of Water Saving Agriculture in Arid Regions of China, Northwest A&F University, Xianyang, China

**Keywords:** *Rht12*, *Ppd-D1a*, flowering time, plant height, yield components, wheat

## Abstract

The gibberellin-responsive dwarfing gene *Rht12* can significantly reduce plant height without changing seedling vigor and substantially increase ear fertility in bread wheat (*Triticum aestivum.* L). However, *Rht12* delays heading date and anthesis date, hindering the use of *Rht12* in wheat improvement. To promote early flowering of the *Rht12* dwarf plants, the photoperiod-insensitive allele *Ppd-D1a* was introduced through a cross between Jinmai47 (*Ppd-D1a*) and Karcagi (*Rht12*). The results showed that *Ppd-D1a* can rescue the delaying effect of *Rht12* on flowering time and promote earlier flowering by 9.0 days (163.2°Cd) in the *Rht12* dwarf plants by shortening the late reproduction phase. Plant height was reduced by *Rht12* (43.2%) and *Ppd-D1a* (10.9%), achieving dwarf plants with higher lodging resistance. Ear fertility, like the grain number per spike, was significantly increased by *Rht12* (21.3%), while it was reduced by *Ppd-D1a* (6.5%). However, thousand kernel weight was significantly reduced by *Rht12* (12.9%) but significantly increased by *Ppd-D1a* (16.9%). Finally, plant yield was increased by 16.4 and 8.2%, and harvest index was increased by 24.9 and 15.4% in the *Rht12* dwarf lines and tall lines with *Ppd-D1a*, respectively. Clearly, there was an additive interaction between *Rht12* and *Ppd-D1* and the introduction of *Ppd-D1a* advanced the flowering time and improved the yield traits of *Rht12* dwarf plants, suggesting that the combination of *Rht12* and *Ppd-D1a* would be conducive to the successful use of *Rht12* in wheat breeding programs.

## Introduction

The introduction of semi-dwarf genes (*Rht-B1b* and *Rht-D1b*) into bread wheat (*Triticum aestivum.* L) was a crucial component of the Green Revolution in the 1970s (Flintham et al., 1997; Hedden, 2003). The higher yields of these semi-dwarf varieties were associated with improved lodging resistance and the resulting ability to tolerate higher rates of chemical fertilizers (Worland et al., 1994). The decrease in stem stature resulted in an increase in assimilate partitioning to developing ears, enabling greater floret survival at anthesis and increased grain numbers per ear (Youssefian et al., 1992). *Rht-B1b* and *Rht-D1b* reduce stem internode length and therefore overall plant height by decreasing the sensitivity of plant tissues to endogenous Gibberellin Acid (GA) (Peng et al., 1999; Sun, 2010). To date, *Rht-B1b* and *Rht-D1b* are still the major semi-dwarf genes in wheat breeding, and there are only a few dwarf genes (*Rht-B1b*, *Rht-D1b*, and *Rht8*) successfully applied in wheat breeding projects (Ellis et al., 2004; Botwright et al., 2005; Zhang et al., 2006). Owing to the narrowed genetic background of dwarfing genes, there is a need to broaden dwarf (*Rht*) genetic resources for wheat production in the future. In the deep sowing conditions, especially in water-limited areas, the semi-dwarf alleles *Rht-B1b* and *Rht-D1b* reduce coleoptile length, seedling vigor, and the capacity to emerge at the seedling stage, leading to low yield and poor final plant biomass (Rebetzke et al., 2001, 2004, 2007; Botwright et al., 2005). In irrigated and fertilized environments, the height reduction effect of *Rht-B1b* and *Rht-D1b* may not be sufficient, and serious lodging could still happen in wheat varieties with these semi-dwarf alleles (Stapper and Fischer, 1990; Berry et al., 2007). Therefore, it is important to include more height reduction genes such as the GA-responsive dwarf gene *Rht12* for wheat breeding (Rebetzke et al., 2012a; Chen et al., 2013).

*Rht12* is located on the long arm of chromosome 5A and can significantly reduce plant height by approximately 40% without impacting coleoptile length and seedling vigor. Furthermore, *Rht12* significantly increased floret fertility, grain number per spike, grain yield, and harvest index (Rebetzke et al., 2012a; Chen et al., 2013). Unfortunately, *Rht12* delays ear emergence time (as well as flowering time) by approximately 6 days, and this adverse effect was observed in many environments (Worland et al., 1994; Chen et al., 2013), limiting the use of *Rht12* in wheat improvement. It was previously reported that the dominant vernalization gene *Vrn-B1*, which promotes earlier flowering, could not rescue the delaying effects of *Rht12* on flowering time, indicating that *Rht12* might be epistatic to *Vrn-B1* (Chen et al., 2013). Therefore, spike development-related genes should be introduced to promote earlier flowering of the *Rht12* dwarf lines for exploiting the potential of *Rht12* in wheat breeding.

The photoperiod genes, which are responsible for photoperiod insensitivity in wheat, play a major role in spike development and flowering time. Genes allelic to *Ppd-1* are located on the homologous group 2 chromosomes (Law et al., 1978). The dominant alleles of photoperiod genes (*Ppd-D1*, *Ppd-B1*, and *Ppd-A1*) showed insensitivity to daylength and accelerated earlier flowering in wheat (Worland et al., 1998). *Ppd-D1a*, the dominant allele of *Ppd-D1*, which is a member of the pseudo response regulator (*PRR*) gene family, is the major source of photoperiod insensitivity in wheat cultivars worldwide and can promote earlier ear emergence and flowering compared to its recessive allele *Ppd-D1b* (Worland et al., 1988; Gonzalez et al., 2005; Wilhelm et al., 2013; Grogan et al., 2016). It was found that allele *Ppd-D1a* was associated with a 2089-bp deletion in the promoter region that altered the expression of the gene, specifically a loss of its normal circadian regulation. The early flowering phenotype of the *Ppd-D1a* mutation is likely to be caused by this alteration and the resultant induction of the key floral regulator flowering locus T (FT) in short or long days (Beales et al., 2007).

The objective of this work was to analyze whether the *Ppd-D1a* allele could rescue the delayed effects of *Rht12* on plant development and promote earlier flowering in the *Rht12* dwarf lines and to explore the interactive effects between *Ppd-D1a* and *Rht12* on plant height and other agronomic traits.

## Materials and Methods

### Plant Materials

A cross was made between two winter bread wheat cultivars Jinmai47 and Karcagi (12). Karcagi (12) is the *Rht12* donor (a gamma ray-induced mutant) carrying the photoperiod-sensitive allele *Ppd-D1b* as detected by molecular markers (2D-Ins-F1; 2D-Ins-R1/R2) described in Beales et al. (2007). Jinmai47 is a Chinese winter wheat cultivar, widely used in the northwest winter wheat region of China, carrying the photoperiod-insensitive allele *Ppd-D1a* and the semi-dwarf allele of *Rht8* (Wang et al., 2015), which was reported at a distance of 21.7 cM from *Ppd-D1* (Gasperini et al., 2012; Chebotar et al., 2013; Wang et al., 2015). Jinmai47 and Karcagi (12) lack any known dominant vernalization genes as detected by molecular markers (VRN1AF, VRN1R; Intr1/A/F2, Intr1/A/R3; Intr1/C/F, Intr1/AB/R; Intr1/B/F, Intr1/B/R3; Intr1/B/F, Intr1/B/R4; Intr1/D/F, Intr1/D/R3; Intr1/D/F, Intr1/D/R4) described in Supplementary Table 1 (Yan et al., 2004; Fu et al., 2005). Additionally, there was no other known dwarfing genes, such as *Rht-B1b*, *Rht-D1b*, *Rht4*, *Rht5*, or *Rht13*, etc., in Jinmai47 and Karcagi (12) detected by molecular markers as described in Supplementary Table 1 (Ellis et al., 2002, 2005).

A total of 306 F_2_ individual lines were derived from the two parents. Based on the corresponding molecular markers of *Rht12* (SSR marker: WMS291) (Korzun et al., 1997) and *Ppd-D1* (STS marker: 2D-Ins-F1/R1/R2) (Beales et al., 2007), we only selected four homozygous genotypes in the F_2_ population, which included 18 lines sharing both the dwarf allele *Rht12* and the photoperiod-insensitive allele *Ppd-D1a* (*Rht12Rht12Ppd-D1aPpd-D1a*, RRPP), 15 lines with the dwarf allele of *Rht12* and the photoperiod-sensitive allele *Ppd-D1b* (*Rht12Rht12Ppd-D1bPpd-D1b*, RRpp), 16 lines with *Ppd-D1a* and *rht12* (*rht12rht12Ppd-D1aPpd-D1a*, rrPP), and 13 lines with *Ppd-D1b* and *rht12* (*rht12rht12Ppd-D1bPpd-D1b*, rrpp). These homozygous plants were used to develop the F_2:3_, F_3:4_, and F_4:5_ lines for further analysis.

### Field Experiments

The two parents and the selected 62 lines were planted at the experimental farm of Northwest A&F University, Xianyang, China (34°17′ N, 108°04′ E, at an elevation of 506 m) in the fall of 2014, 2015, and 2016, respectively. Each experiment was conducted under natural photoperiod conditions with the average daylength ranging from 12.6 to 13.5 h at the late reproductive phase in the experimental farm. To avoid competitive effects between the tall and dwarf plants, the 33 dwarf lines were randomly arranged in a dwarf plot, and the 29 tall lines were randomly arranged in a separate tall plot. Each experiment was conducted with two replicates. Experimental plots were 1.0 m × 1.5 m, with four rows spaced 25 cm apart. Seeds were planted at a distance of 5 cm between seeds within rows. Supplemental irrigation was provided as needed for avoiding water stress. Insecticides and fungicides were used to prevent insect and disease damage. Weather data were collected by the weather station at this experimental field.

### DNA Extraction and Genotyping

The genomic DNA of the F_2_ individuals and the two parents were extracted by using the CTAB method (Porebski et al., 1997). The presence of *Rht12* was detected with the SSR marker WMS291 (Xgwm291) and the presence of *Ppd-D1* was checked with STS markers 2D-Ins-F1/R1/R2 in the F_2_ population, respectively, as described (Korzun et al., 1997; Beales et al., 2007; Chen et al., 2013). Markers were purchased from AuGCT Biotech Co., Ltd, Beijing. Then, the homozygous genotypes at these two loci were selected to develop the F_2:3_, F_3:4_, and F_4:5_ lines.

### Spike Development and Fertility

Spike differentiation was investigated every 4 or 7 days using the main shoot of the sampled plants from the five-leaf stage (Z15), for checking the timing of the double ridge formation stage (DR) and the terminal spikelet initiation stage (TS) (Gardner et al., 1985). Three plants were selected from each plot every time and a digital stereo microscope (Nikon, SMZ1500) linked with a digital camera was used for observing and taking pictures. The anthesis dates (Z65) of each line were recorded as that 50% of the spikes in a plot flowered (Gonzalez et al., 2005; Chen et al., 2013). Thermal time was used for evaluating the progress of spike development. Thermal time was evaluated by the accumulated growing degree day (°Cd), which was recorded as the sum of the daily mean temperature above a base temperature suitable for plant growth from the sowing date through the observed dates to the target phases; here, 0°C was selected as the base temperature (Shaykewich, 1995; Gonzalez et al., 2005; Ibrahim et al., 2009).

At anthesis, the main shoot spike of five plants from each plot were randomly selected to count the number of fertile florets. Stigmatic branches of florets spread wide with either green anthers or with pollen grains present on them were considered fertile (Waddington et al., 1983).

### Plant Height and Internode Characteristics

Five plants per plot were randomly selected for measuring the leaf number of the main shoot (Haun, 1973). Peduncle length (cm) and plant height (cm) were measured at maturity. Lodging was scored at maturity from the fraction of the area affected and the severity of lodging in those areas. This degree of lodging is judged as the angle from the vertical. That is, going from 0 for a standing crop to 90 for a crop flat on the ground. The lodging score is then the proportion of the area affected multiplied with the degree of lodging (Zadoks et al., 1974).

### Yield and Yield Components

Ten plants were randomly selected per plot for collecting the data of grain number per spike, spikelet number per spike, and fertile shoot number per plant at maturity. Before the harvest, approximately 20 to 45 plants remained in each line because of the frequent sampling, and these plants were used for measuring the biomass per plant, grain yield per plant, harvest index, and thousand kernel weight (for details see Chen et al., 2013).

### Data Analysis

The mean values of all the investigated traits for the four genotypes (RRPP, RRpp, rrPP, and rrpp) were calculated and statistical analysis was carried out by analysis of variance (ANOVA) using the statistical package SPSS 18.0, as outlined in previous studies (Liu et al., 2017; Chen et al., 2018). ANOVA was initially performed on the yearly data and combined environments over the 3 years. Each genotype was considered as a fixed effect to calculate mean, while the replication was treated as a random effect in each year. For the combined model, year was also considered as a random effect. Pairwise multiple comparisons were detected using a protected least significant differences (LSD) test at α = 0.05. The relative effects of dwarf genes were estimated by using the formula: effect = (Mean_dwarf_ – Mean_tall_)/Mean_tall_ × 100%.

## Results

### Molecular Marker Detection of *Rht12* and *Ppd-Da*

**FIGURE 1:**
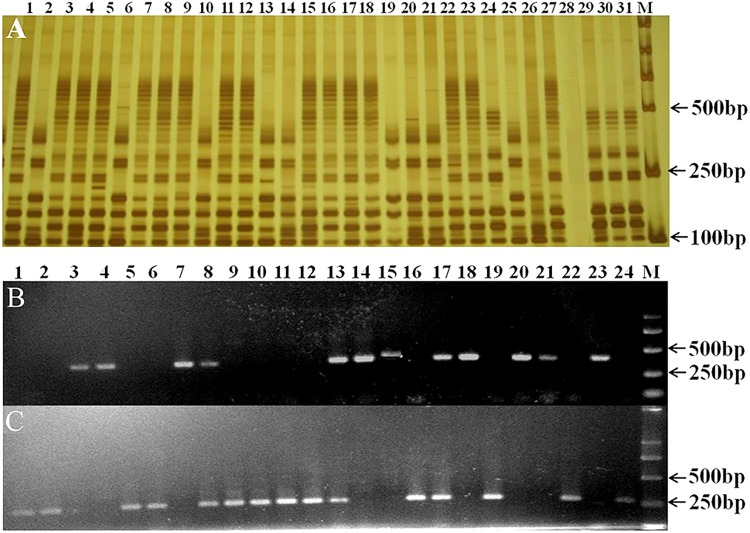
Detection of the dwarf gene *Rht12* and photoperiod gene *Ppd-D1* with SSR marker WMS291 **(A)** and STS markers 2D-Ins-F1/R1/R2 **(B,C)** in some plants from the F_2_ population. **(A)** 24, 29, 30, 31, homozygous dwarfing individuals (*Rht12Rht12*); 2, 6, 10, 13, 14, 19, 20, 21, 25, homozygous tall individuals (*rht12rht12*); 28, no amplification observed; the others were heterozygous individuals (*Rht12rht12*). **(B,C)** 1, 2, 5, 6, 9, 10, 11, 12, 16, 19, 22, 24, homozygous individuals with *Ppd-D1a*; 3, 4, 7, 14, 15, 18, 20, 21, 23, homozygous individuals with *Ppd-D1b*; the others were heterozygous individuals (*Ppd-D1a Ppd-D1b*). M: marker DL2000.

The homozygous genotypes of *Rht12* and *Ppd-D1* in the F_2_ segregating population were classified based on the presence or absence of their linked SSR and STS markers (Figure 1). Homozygous individuals at these two loci were selected and used to develop the F_2:3_, F_3:4_, and F_4:5_ lines for further analysis.

### ANOVA of All Traits Among Parents and the Four Genotypes

On average, the taller parent Jinmai47 (rrPP) was 20.7 cm higher than the dwarf donor parent Karcagi (RRpp) over the 3 years. Jinmai47 also showed less grain number per spike but better yield and yield-associated traits than Karcagi. Transgressive segregation was found in the population for all traits and all the traits were significant (*P* < 0.01) from 2014 to 2016 except florets initiated per spikelet (*P* = 0.4) and florets initiated per spike (*P* = 0.03) (Table 1). Parent Jimmai47 shared the same genotype with 16 lines for the *Ppd-D1* and *Rht12* genes. Jinmai47 was 18.3 cm shorter than the progenies and had a shorter peduncle length with a better lodging score. In addition, 15 lines had the same genotype with Karcagi, and the progenies were similar on the traits we recorded but better on fertility (Table 1).

**Table 1:** Mean and analysis of variance of all traits among parents and the four genotypes on *Rht12* and *Ppd-D1* during 2014–2016.

Traits	Parents			Population (*n* = 62)					
	Jinmai47	Karcagi	Diff.	RRPP	RRpp	rrPP	rrpp	*F*-value	*P*-value
Plant height (cm)	89.5	68.8	20.7	60.8	69.0	107.8	119.3	213.6	<0.0001
peduncle length (cm)	26.7	23.8	2.8	21.6	24.1	35.2	42.1	7.1	0.0001
Lodging score	1.7	10.0	-8.3	0.0	1.7	23.3	50.0	5.3	<0.0001
Thermal time (°Cd) from sowing to anthesis	1448.3	1834.0	-385.7	1530.2	1693.4	1483.8	1663.9	556.5	<0.0001
Spikelets spike^-1^	20.7	19.7	1.0	19.0	19.5	20.4	21.1	43.2	<0.0001
Florets initiated spikelet^-1^	9.0	9.5	-0.5	9.3	9.5	9.5	9.7	0.9	0.4128
Florets initiated spike^-1^	199.2	190.2	9.0	176.1	180.0	192.2	197.7	2.3	0.0301
Fertile florets spike^-1^	52.0	55.0	-3.0	49.7	54.9	45.3	48.8	113.0	<0.0001
Fertility^∗^ (%)	25.2	28.3	-3.1	28.2	30.6	23.7	25.5	62.9	<0.0001
Spike length (cm)	10.4	12.9	-2.6	9.9	10.4	10.7	12.4	8.0	<0.0001
Efficient spikes plant^-1^	6.0	9.4	-3.4	7.6	7.5	6.5	5.9	8.3	<0.0001
Grain No. spike^-1^	47.6	50.1	-2.5	42.7	44.5	39.3	41.0	12.4	<0.0001
TKW (g)	48.0	35.0	13.0	41.9	35.4	45.7	42.3	37.6	<0.0001
Yield (g)	15.6	11.6	4.0	14.4	12.6	15.4	14.4	16.2	<0.0001
Biomass (g)	40.4	34.4	6.0	35.0	38.0	41.3	44.0	18.5	<0.0001
Harvest index (%)	40.5	33.3	7.2	40.3	32.5	37.8	32.8	28.4	<0.0001

^∗^*Fertility is estimated as the ratio of fertility florets to total florets per spike of the main shoot spike*.

### Plant Height and Associated Traits

**FIGURE 2:**
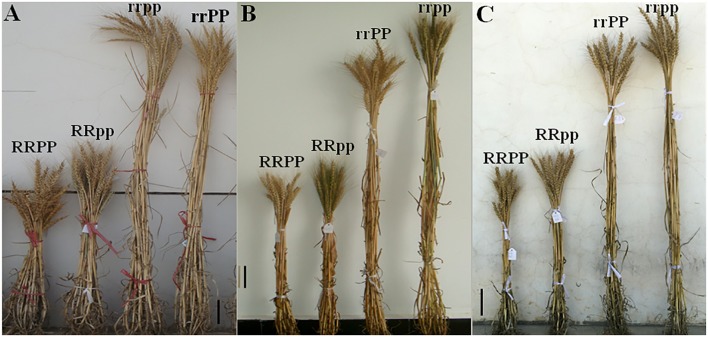
Plant morphology of the four groups of the F_2:3_, F_3:4_, and F_4:5_ lines at maturity. **(A–C)** Plant morphology of the four groups of the F_2:3_, F_3:4_, and F_4:5_ lines in the 2014–2015, 2015–2016, and 2016–2017 growing seasons, respectively. Scale bar = 10 cm.

The plant height of the dwarf lines (RR) was significantly shorter than the tall lines (rr) by an average of 44.1 cm (41.3%), 51.8 cm (44.6%), and 52.6 cm (43.7%) in F_2:3_, F_3:4_, and F_4:5_ (Table 2), respectively. However, in the dwarf lines (RR), the plant height of RRPP was reduced by 7.6 cm (11.1%) on average compared with that of the RRpp lines across the three generations. In the tall lines (rr), the plant height of the rrPP was reduced by 12.9 cm (10.7%) on average compared with that of the rrpp lines across the three generations (Table 2 and Figure 2). It is clear that the dominant *Ppd-D1a* allele decreases plant height in both the dwarf and tall lines, though its effect is much weaker than that of *Rht12*.

**Table 2:** Plant height and lodging score of the four groups of wheat lines in the 3-year experiments.

Progeny	Traits	RRPP	RRpp	rrPP	rrpp	Jinmai47	Karcagi
F_2:3_ 2014–2015	Plant height (cm)	58.3 ± 5.6d	67.2 ± 4.7c	98.1 ± 6.5b	115.7 ± 7.0a	89.7 ± 2.2	68.6 ± 3.2
	Peduncle length (cm)	19.5 ± 2.4d	22.7 ± 3.0c	32.6 ± 3.3b	40.0 ± 4.1a	25.5 ± 1.3	23.4 ± 2.8
	Lodging score (%)	0.0	0.0	20.0	45.0	0.0	0.0
F_3:4_ 2015–2016	Plant height (cm)	60.8 ± 3.9d	67.6 ± 5.0c	110.2 ± 5.0b	121.7 ± 5.8a	91.4 ± 3.1	71.5 ± 2.8
	Peduncle length (cm)	22.8 ± 2.0d	25.5 ± 2.5c	35.7 ± 2.7b	42.5 ± 3.6a	26.2 ± 1.4	24.8 ± 2.0
	Lodging score (%)	0.0	5.0	45.0	90.0	10.0	0.0
F_4:5_ 2016–2017	Plant height (cm)	64.2 ± 4.5d	71.3 ± 4.3c	115.6 ± 5.1b	125.1 ± 5.5a	91.7 ± 2.0	73.0 ± 3.8
	Peduncle length (cm)	20.7 ± 1.6d	23.2 ± 3.3c	36.6 ± 4.0b	45.5 ± 3.4a	27.8 ± 1.2	24.1 ± 3.0
	Lodging score (%)	0.0	0.0	5.0	30.0	5.0	0.0

*All data are represented as mean ± SD of each genotype. Different letters (“a,” “b,” “c,” and “d”) within rows indicate differences significant between different genotypes at *P* < 0.05; parents were not included*.

The culm of tall lines elongated faster than that of *Rht12* dwarf lines from the seedling stage, and the final plant stature of *Rht12* dwarf lines was shortened through the reduction of the length of the internodes, especially the reduction of the peduncle length. The peduncle length of the RR lines was reduced by 16.3 cm (42.0%) on average compared to that of the tall lines (rr) across the three generations (Table 2). Additionally, the *Ppd-D1a* allele showed a reducing effect on peduncle length that was much weaker than that of *Rht12*. The peduncle length of the RRPP group was shorter than that of the RRpp group by 2.8 cm (11.8%); the peduncle length of the rrPP group was shorter than that of the rrpp group by 7.7 cm (18.0%) across the three successive generations, respectively (Table 1). The plant height was reduced by either *Rht12* or *Ppd-D1a* through a reduction of the length of each internode instead of a reduction of the number of internodes (both the tall and dwarf plants have 5 internodes, data not shown). Finally, the dwarf plants had greater resistance to lodging, probably due to the dwarf stature and the shorter internodes, whereas serious lodging only occurred in the tall lines.

### Spike Development and Flowering Time

For all the three generations of progeny, the duration of the sowing – double ridge formation stage (SW-DR) of the *Rht12* dwarf lines was longer than that of the tall lines, while there was no significant difference between the RRPP and RRpp, and the rrPP and rrpp groups (Table 3). Compared to the tall lines (rr) in F_2:3_, F_3:4_, and F_4:5_, the dwarf (RR) lines require an additional 45.7°Cd (7.0 days), 32.2°Cd (7.0 days), and 47.6°Cd (6.5 days), respectively, to reach the double ridge stage. These results indicated that *Ppd-D1a* did not significantly affect the duration of the vegetative phase (SW-DR period), though the lines with *Ppd-D1a* had a shorter duration of the SW-DR period than the lines with *Ppd-D1b*. Finally, due to the longer duration of the vegetative phase (SW-DR period), the dwarf plants produced more leaves than the tall plants in all the three generations of progeny (Table 3).

**Table 3:** Thermal time (°Cd) and duration days (d) of different developmental phases of the four genotypes of the F_2:3_, F_3:4_, and F_4:5_ lines.

Progeny	Genotype/ variety	SW-DR	DR-TS	TS-AN	SW-AN	Total leaf number
F_2:3_ 2014–2015	RRPP	779.6(155.0)a	187.2(16.0)b	594.8(41.0)c	1561.6(212.0)c	13.1a
	RRpp	790.6(156.0)a	204.2(17.0)a	779.9(48.0)a	1714.9(221.0)a	13.6a
	rrPP	731.8(147.0)b	156.0(18.0)c	609.4(43.0)c	1497.2(208.0)d	12.0b
	rrpp	746.9(150.0)b	189.7(19.0)b	745.8(50.0)b	1682.4(219.0)b	12.0b
	Karcagi	857.3(162.0)	230.4(18.0)	795.3(49.0)	1883.0(229.0)	15.3
	Jinmai47	724.1(145.0)	133.2(17.0)	610.7(44.0)	1468.0(206.0)	11.7
F_3:4_ 2015–2016	RRPP	788.3(148.0)a	162.2(19.0)c	566.0(41.0)c	1516.5(208.0)c	12.5a
	RRpp	797.1(150.0)a	207.7(23.0)a	672.1(44.0)a	1676.9(217.0)a	12.2a
	rrPP	750.5(140.0)c	143.2(22.0)d	587.1(44.0)c	1480.8(206.0)d	11.2b
	rrpp	770.5(144.0)b	180.0(23.0)b	711.4(49.0)b	1661.9(216.0)b	11.5b
	Karcagi	837.4(157.0)	248.2(22.0)	726.3(45.0)	1811.9(224.0)	16.0
	Jinmai47	746.7(139.0)	131.7(21.0)	559.4(44.0)	1437.8(204.0)	10.2
F_4:5_ 2016–2017	RRPP	796.9(150.0)a	163.0(18.0)b	552.7(38.0)c	1512.6(206.0)c	13.3a
	RRpp	836.3(154.0)a	203.9(22.0)a	648.2(39.0)a	1688.4(215.0)a	13.5a
	rrPP	755.9(145.0)b	153.6(19.0)c	554.1(39.0)c	1473.5(204.0)d	12.3b
	rrpp	782.2(148.0)b	200.5(22.0)a	664.8(43.0)b	1647.5(213.0)b	12.2b
	Karcagi	864.6(159.0)	238.3(21.0)	706.2(42.0)	1809.1(222.0)	15.1
	Jinmai47	748.2(144.0)	150.3(19.0)	540.6(39.0)	1439.1(202.0)	11.0

*Data are the calculated thermal time (°Cd) with duration days (d) in brackets; SW, sowing date; DR, double ridge formation date; TS, terminal spikelet initiation date; AN, anthesis date. Data are shown as the mean of each group. Thermal time was used for statistical analysis. Different letters (“a,” “b,” “c,” and “d”) within the same column represent differences significant at *P* < 0.05; parents were not included*.

**FIGURE 3:**
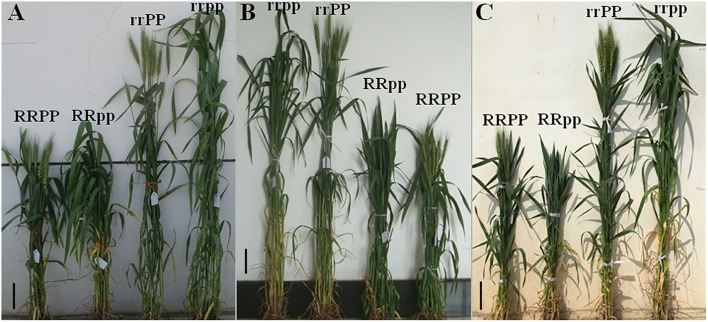
Plant morphology of the four groups (RRPP, RRpp, rrPP, and rrpp) of the F_2:3_, F_3:4_, and F_4:5_ lines at the heading (or anthesis) stage. **(A–C)** Plant morphology of the four groups of wheat F_2:3_, F_3:4_, and F_4:5_ lines in the 2014–2015, 2015–2016, and 2016–2017 growing seasons, respectively. The RRPP and rrPP plants have headed and some plants have even flowered, while the RRpp and rrpp plants have just reached the heading stage, and some plants are still at the late booting stage. Scale bar = 10 cm.

However, after the vegetative phase, *Ppd-D1a* showed a significant effect on the subsequent developmental phases, double ridge stage (DR) to TS and to anthesis stage (AN). Actually, quantitative trait loci (QTL) analysis was conducted using this F_2_ population (306 plants). Results revealed that the locus of *Ppd-D1* explained approximately 70.0% of the flowering time variation, indicating that *Ppd-D1* is a major locus for flowering time in this population. RRPP lines required less thermal time to complete the DR-TS period and reached the TS stage 34.5°Cd on average earlier than the RRpp lines across F_2:3_, F_3:4_, and F_4:5_ (Table 3). Additionally, *Ppd-D1a* accelerated the developmental progress of the reproductive phase in the tall lines. The duration of the DR-TS period of the rrPP group was 39.1°Cd on average shorter than that of the rrpp group across the three generations. Moreover, for the duration of TS-AN period, RRPP had shorter TS-AN period by 128.9°Cd than the RRpp group, whereas the rrPP group showed a shorter duration of TS-AN than the rrpp group by 123.8°Cd across the three generations. These results indicated that either the dwarf or tall plants carrying *Ppd-D1a* had a shorter duration of DR-AN period. Finally, the RRPP and rrPP lines flowered earlier by 163.2°Cd (9.0 days) and 180.1°Cd (10.0 days) on average than the RRpp and rrpp lines, respectively, across the three generations (Table 3 and Figure 3). It was therefore clear that the reproductive phase can be strongly affected by *Ppd-D1a* in both the dwarf and tall plants, without significantly affecting the vegetative phase.

### Floret Fertility

In the three generations, the number of spikelets per spike of the dwarf lines (RRPP and RRpp) was significantly lower by 1.5 (7.3%) on average than the tall lines (rrPP and rrpp), while no significant difference was observed between the PP and pp genotypes (Table 4). However, there was no significant difference observed between the four genotypes in the number of florets initiated per spikelet. Generally, 9 to 10 florets could be initiated per spikelet. Finally, the tall plants achieved larger numbers of florets per spike than the dwarf plants owing to their larger number of spikelets per spike. Additionally, the pp plants had more florets per spike than the PP plants in all the three generations (Table 4). *PPd-D1b* likely played a major role in determining more floret numbers in both the tall and dwarf lines.

**Table 4:** Spike characters of the four genotypes of the F_2:3_, F_3:4_, and F_4:5_ lines.

Progeny		Genotype/variety					
		RRPP	RRpp	rrPP	rrpp	Jinmai47	Karcagi
F_2:3_ (2014–2015)	Spikelets spike^-1^	19.2 ± 1.3a	19.6 ± 1.5a	20.7 ± 1.0b	21.0 ± 1.7b	22.3 ± 1.6	19.0 ± 1.0
	Florets initiated spikelet^-1^	9.5 ± 1.3a	10.0 ± 1.1a	9.4 ± 0.8a	9.8 ± 1.0a	9.3 ± 1.0	9.4 ± 1.2
	Florets initiated spike^-1^	186.4 ± 5.0c	193.0 ± 4.6b	200.1 ± 7.1a	206.2 ± 6.2a	210.1 ± 5.4	183.7 ± 6.0
	Fertile florets spike^-1^	52.0 ± 1.8b	58.1 ± 2.0a	47.0 ± 1.6c	52.1 ± 2.2b	53.6 ± 2.4	57.0 ± 2.8
	Fertility^∗^	0.28 ± 0.01b	0.31 ± 0.01a	0.23 ± 0.01d	0.25 ± 0.01c	0.26 ± 0.01	0.31 ± 0.02
F_3:4_ (2015–2016)	Spikelets spike^-1^	19.0 ± 1.0a	19.3 ± 1.2a	20.2 ± 1.5b	20.6 ± 1.0b	21.0 ± 1.2	20.0 ± 1.3
	Florets initiated spikelet^-1^	9.1 ± 0.5a	9.0 ± 1.0a	9.5 ± 0.8a	9.2 ± 1.1a	9.6 ± 0.6	8.9 ± 0.5
	Florets initiated spike^-1^	171.0 ± 3.2c	176.1 ± 5.0c	183.6 ± 4.5b	190.7 ± 3.3a	200.1 ± 6.0	181.4 ± 5.1
	Fertile florets spike^-1^	47.2 ± 2.0b	52.1 ± 2.3a	42.2 ± 2.4d	45.0 ± 3.0c	50.0 ± 3.4	53.1 ± 4.1
	Fertility^∗^	0.28 ± 0.01b	0.30 ± 0.02a	0.23 ± 0.01d	0.24 ± 0.01c	0.25 ± 0.01	0.29 ± 0.01
F_4:5_ (2016–2017)	Spikelets spike^-1^	19.0 ± 1.5a	19.5 ± 1.0a	20.8 ± 1.6b	21.3 ± 1.2b	21.2 ± 1.4	19.4 ± 1.1
	Florets initiated spikelet^-1^	9.0 ± 0.5b	9.7 ± 1.0a	9.6 ± 1.0a	9.9 ± 0.7a	9.0 ± 1.0	9.5 ± 0.6
	Florets initiated spike^-1^	170.3 ± 4.2d	177.6 ± 5.3c	192.5 ± 4.6b	197.1 ± 5.5a	190.5 ± 5.0	195.4 ± 4.3
	Fertile florets spike^-1^	50.0 ± 3.2b	55.1 ± 4.0a	47.6 ± 3.7c	50.5 ± 2.6b	49.5 ± 2.9	55.0 ± 3.6
	Fertility^∗^	0.29 ± 0.01b	0.31 ± 0.01a	0.25 ± 0.01d	0.26 ± 0.01c	0.26 ± 0.01	0.28 ± 0.01

^∗^*Fertility is estimated as the ratio of fertility florets to total florets per spike of the main shoot spike. All data are the mean of each genotype. Different letters (“a,” “b,” “c,” and “d”) within rows indicate differences significant at *P* < 0.05; parents were not included*.

The dwarf plants produced 5.5 (11.0%), 6.1 (13.9%), and 3.5 (7.1%) more fertile florets per spike than the tall plants in F_2:3_, F_3:4_, and F_4:5_, respectively. Additionally, the pp genotype achieved more fertile florets per spike than the PP genotype by 10.7% in the dwarf lines and 7.9% in the tall lines, among the three generations (Table 4). Finally, the ear fertility of the RR groups was significantly increased by 22.9, 23.4, and 17.6% than that of the rr groups in the three generations, respectively. Additionally, RRpp achieved higher fertility than RRPP, and rrpp also had higher fertility than rrPP in the three generations. This suggested that both *Rht12* (RR) and *PPd-D1b* (pp) could increase ear fertility, and the combination of RR and pp had a stronger potential in improving floret fertility than the other three genotypes.

### Yield and Yield Components

The spike length of RR was shorter than that of rr by an average of 8.2%, across the three generations. Moreover, in the dwarf lines, the spike length of RRpp was longer by an average of 1.4 cm (13.8%) than that of RRPP; in the tall lines, the spike length of rrpp was longer by an average of 1.7 cm (15.6%) than that of rrPP in the three generations (Table 5). It was indicated that the *Ppd-D1a* allele and *Rht12* would decrease spike length. The number of spikelets per spike of the pp plants was larger than that of the PP plants in both the dwarf and tall groups, while *Rht12* only had a weak effect. However, the RR genotype produced more efficient spikes (1.37; 22.2%) than the rr genotype, while no significant difference was observed between the PP and pp genotypes in the three generations, suggesting that *Rht12* has a positive effect on this trait. Grain number per spike of the *Rht12* dwarf lines (RR) was significantly higher than that of the tall lines (rr) by an average of 3.5 (8.6%). Additionally, pp plants also showed a stronger effect on grain number per spike; pp plants had more grains per spike than PP plants in both the dwarf (5.2%) and tall groups (5.0%) (Table 5), indicating that *Rht12* and *Ppd-D1b* both contributed to producing more grains per spike.

**Table 5:** Yield-related traits of the four genotypes of the F_2:3_, F_3:4_, and F_4:5_ lines.

Progeny	Genotype/ variety	Spike length (cm)	Spikelet spike^-1^	Number of efficient spikes plant^-1^	Grain No. spike^-1^	Thousand kernel weight (g)	Plant yield^∗^ (g)	Plant biomass^∗^ (g)	Harvest index
F_2:3_	RRPP	9.9 ± 0.4c	19.0 ± 0.5b	8.5 ± 3.1a	44.0 ± 3.9b	41.6 ± 2.7b	14.3 ± 1.9a	34.2 ± 3.7c	0.42 ± 0.04a
2014–2015	RRpp	11.2 ± 0.7b	19.6 ± 0.8a	8.0 ± 3.0a	46.1 ± 3.2a	37.3 ± 2.7c	13.0 ± 1.9b	36.0 ± 4.3c	0.36 ± 0.03c
	rrPP	10.8 ± 0.6b	19.0 ± 1.0b	6.6 ± 2.2b	39.5 ± 3.3d	45.4 ± 2.9a	15.1 ± 3.0a	40.2 ± 4.2b	0.38 ± 0.03b
	rrpp	12.4 ± 1.0a	20.0 ± 1.0a	7.0 ± 2.4b	42.2 ± 3.5c	42.2 ± 3.3b	14.6 ± 3.0a	42.5 ± 5.6a	0.34 ± 0.03c
	Karcagi	12.3 ± 1.1	20.0 ± 1.2	9.9 ± 2.4	49.6 ± 4.2	34.1 ± 2.8	12.3 ± 1.8	33.9 ± 4.8	0.36 ± 0.04
	Jinmai47	9.9 ± 0.3	20.5 ± 0.6	6.2 ± 1.8	46.8 ± 4.2	46.2 ± 3.0	15.7 ± 1.9	41.7 ± 4.5	0.39 ± 0.03
F_3:4_	RRPP	9.7 ± 0.5d	18.5 ± 1.0c	6.3 ± 2.2a	40.3 ± 3.5b	40.2 ± 3.3b	13.8 ± 1.2a	35.0 ± 3.6d	0.39 ± 0.02a
2015–2016	RRpp	11.5 ± 1.0b	19.5 ± 1.0b	6.6 ± 1.8a	42.6 ± 4.1a	34.5 ± 3.6c	11.4 ± 1.6b	38.6 ± 3.3c	0.30 ± 0.03c
	rrPP	10.6 ± 0.6c	18.6 ± 0.5c	5.4 ± 1.0b	38.7 ± 3.8c	44.3 ± 4.1a	15.4 ± 2.1a	41.3 ± 3.8a	0.37 ± 0.03b
	rrpp	12.7 ± 0.4a	20.6 ± 0.7a	5.1 ± 2.0b	39.6 ± 2.6c	40.6 ± 3.4b	14.0 ± 1.7a	43.6 ± 4.0a	0.32 ± 0.03c
	Karcagi	12.6 ± 0.3	19.0 ± 1.0	10.0 ± 3.2	51.0 ± 5.2	33.7 ± 3.8	10.9 ± 2.0	35.8 ± 4.4	0.30 ± 0.03
	Jinmai47	11.2 ± 0.5	20.3 ± 0.6	7.0 ± 1.5	49.3 ± 3.9	48.4 ± 4.0	15.2 ± 2.5	38.1 ± 3.2	0.41 ± 0.03
F_4:5_	RRPP	10.0 ± 0.4d	19.5 ± 0.5b	8.0 ± 2.8a	43.0 ± 5.1b	43.5 ± 2.7c	15.0 ± 2.0a	36.5 ± 2.5d	0.41 ± 0.03a
2016–2017	RRpp	11.0 ± 0.6b	20.0 ± 0.7a	7.7 ± 2.5a	45.0 ± 5.0a	35.5 ± 3.2d	12.7 ± 1.7c	39.6 ± 3.4c	0.32 ± 0.02c
	rrPP	10.6 ± 0.7c	19.0 ± 1.1b	6.6 ± 2.3b	39.0 ± 4.7d	48.3 ± 3.6a	16.0 ± 1.3a	42.5 ± 3.8b	0.38 ± 0.03b
	rrpp	11.9 ± 0.5a	20.5 ± 0.8a	6.2 ± 2.2b	41.3 ± 4.3c	45.1 ± 4.1b	14.4 ± 2.3b	45.6 ± 4.4a	0.32 ± 0.03c
	Karcagi	13.0 ± 1.0	21.0 ± 0.6	9.0 ± 2.4	50.2 ± 4.1	36.2 ± 3.3	11.5 ± 1.8	34.0 ± 3.6	0.34 ± 0.02
	Jinmai47	10.1 ± 0.2	21.5 ± 0.8	6.6 ± 1.6	47.0 ± 4.3	50.5 ± 4.4	16.0 ± 2.0	41.0 ± 3.3	0.41 ± 0.04

^∗^*These data are the mean values of 20–45 plants. All data are mean ± SD of each genotype. Different letters (“a,” “b,” “c,” and “d”) within the same column represent differences significant at *P* < 0.05; parents were not included*.

*Rht12* significantly reduced the thousand kernel weight in both the PP and pp lines in all the three generations. However, *Ppd-D1a* significantly increased the thousand kernel weight in both the dwarf and tall lines. The thousand kernel weight of the PP lines was significantly higher by an average of 6.0 g (16.9%) and 3.4 g (7.9%) than that of the pp lines in the dwarf and tall groups, respectively, across the three generations (Table 5).

In the three generations, the rrPP group produced the highest plant yield, while the RRpp group exhibited the lowest plant yield among the four groups, and no significance was observed between the RRPP and rrPP groups. Additionally, in the dwarf groups, plant yield of RRPP was higher by an average of 2.0 g (9.7%) than that of RRpp across the three generations. In the tall lines, plant yield of rrPP lines was higher by an average of 1.2 g (8.2%) than that of rrpp lines, though it was only significant in F_4:5_. The tall lines (rr) achieved a larger plant biomass than the dwarf lines (RR). Finally, RRPP lines achieved the highest harvest index among the four groups. It was clear that the combination of RR and PP had the potential for increasing plant yield and harvest index (Table 5).

### Additive Interaction Between *Rht12* and *Ppd-D1*

Lines with the dwarf allele at *Rht12* flowered 37.9°Cd later than the tall lines, while lines with the early (photoperiod-insensitive) allele at *Ppd-D1* flowered 171.6°Cd earlier than the late allele (Table 6). However, lines with both alleles (dwarf-early) flowered earlier than the tall-late lines (lines with both the tall allele at *Rht12* and the late allele at *Ppd-D1*) by approximately 133.7°Cd, suggesting that the early allele of *Ppd-D1* can rescue the delaying effect of *Rht12* on flowering time and there were additive interaction effects between *Rth12* and *Ppd-D1* on flowering time. This effect was also observed at different spike development phases. Additionally, lines with the dwarf allele at *Rht12* were 48.7 cm shorter than that with the tall allele, whereas lines with the early (photoperiod-insensitive) allele at *Ppd-D1* were 9.9 cm shorter than that with the late allele. However, lines with both alleles (dwarf-early) showed an even shorter plant height compared with the tall-late lines by approximately 58.5 cm (Table 6), indicating that there were additive interaction effects between *Rth12* and *Ppd-D1* on plant height.

**Table 6:** Comparison of the lines with *Rht12* and *Ppd-D1* to explain the interaction effect for anthesis-associated traits and plant height.

Gene	Genotype	SW-DR (°Cd)	DR-TS (°Cd)	TS-AN (°Cd)	SW-AN (°Cd)	Height (cm)
*Rht12*	dwarf	798.1	188.0	635.6	1611.8	64.9
	tall	756.3	170.5	645.4	1573.9	113.6
	difference	41.8^∗^	17.5^∗^	-9.8^∗^	37.9^∗^	-48.7^∗^
*Ppd-D1*	early	767.2	160.9	577.4	1507.0	84.3
	late	787.3	197.7	703.7	1678.7	94.2
	difference	-20.1^∗^	-36.8^∗^	-126.4^∗^	-171.6^∗^	-9.9^∗^
both	dwarf-early	788.3	170.8	571.2	1530.2	60.8
	tall-late	766.5	190.1	707.3	1663.9	119.3
	difference	21.7^∗^	-19.3^∗^	-136.2^∗^	-133.7^∗^	-58.5^∗^

^∗^*P < 0.05*.

## Discussion

This study is part of a series of efforts to obtain a better understanding of the dwarf gene *Rht12* and to promote its use in wheat improvement (Chen et al., 2013, 2014). In previous studies, the effects of *Rht12* on spike development and a range of agronomic traits were analyzed comprehensively (Worland et al., 1994; Wojciechowski et al., 2009; Rebetzke et al., 2012a; Chen et al., 2013). To improve the late ear emergence and flowering time of cultivars with the dwarf allele *Rht12*, (Worland et al., 1994; Chen et al., 2013), the photoperiod-insensitive allele (*Ppd-D1a*) was introduced in this study. Homozygous F_2:3_, F_3:4_, and F_4:5_ lines on the two genes (RRPP, RRpp, rrPP, and rrpp) were chosen to assess the interactive effects of *Rht12* and *Ppd-D1a* on plant development, plant height, and other agronomic traits and to know whether *Ppd-D1a* could promote spike development in *Rht12* dwarf plants. Although the homozygous F_2:3_, F_3:4_, and F_4:5_ lines may have different backgrounds compared with the near-isogenic lines, the larger number of lines and 3-year replications used in this study could neutralize the effects of other loci and environmental errors to some extent. Good resolution could be achieved due to the strong phenotypic effect of *Rht12*, as well as other dwarf genes, as approved in our previous studies. (Chen et al., 2013, 2014, 2018; Daoura et al., 2014; Wang et al., 2014, 2015; Yang et al., 2015, 2017).

Previous studies showed that *Rht12* could significantly lengthen the duration of sowing to double ridge formation and largely delay the anthesis date, and this delaying effect could not be rescued by the dominant *Vrn-B1* allele (Chen et al., 2013). The *Rht12* dwarf lines needed more time to undergo vernalization and had longer reproductive phases (Chen et al., 2013). Here, it was found that *Ppd-D1a* could not alter the SW-DR period significantly in the dwarf lines, either. However, after the vegetative phase, spike development of the PP lines became faster than that of the pp lines in both the tall and dwarf lines. *Ppd-D1a* could significantly shorten the duration of the DR-TS period, especially the duration of the TS-AN period. Finally, PP plants flowered earlier than the pp lines in both height categories. *Ppd-D1a* rescued the delaying effect of *Rht12* on anthesis date. A similar result was reported by Gonzalez et al. (2005) that the duration of the DR-TS and TS-AN periods were significantly shortened by *Ppd-D1a* under natural field conditions. Moreover, the vegetative phase (SW-DR period) was also shortened by *Ppd-D1a* (Gonzalez et al., 2005), though there was no significant difference observed on this trait in our study. The strong effect of *Rht12* on vernalization requirement may be the main reason of this difference. Actually, flowering is a complicated trait mainly controlled by vernalization genes (*Vrn*), photoperiod genes (*Ppd*), and developmental rate genes (“earliness per se,” *Eps*). Further effect of *Ppd-D1* on spike development should be analyzed in this population. Additionally, the delaying effect on flowering time was probably associated with *Rht12* rather than being caused by *vrn-A1*, though *Rht12* was closely linked with *vrn-A1* on chromosome 5A (Worland et al., 1994; Korzun et al., 1997). In fact, the *Vrn-A1* loci in the two parents (Karcagi 12 and Jinmai47) are all recessive as determined by marker detection, and the dwarf and tall plants in this study probably shared the same *vrn-A1* allele. Therefore, the delaying effect of *Rht12* on flowering time might be independent of *vrn-A1*. Exogenous GA_3_ could significantly shorten the duration of the SW-DR period as well as other preanthesis phases in the dwarf lines; exogenous GA_3_ rescued the masking effect of *Rht12* on *Vrn-B1*, which made the dwarf plants with *Vrn-B1* display a nearly spring phenotype (Chen et al., 2014). Moreover, exogenous GA_3_ also restored other dwarf characteristics of *Rht12* to normal, suggesting that the *Rht12* mutant may be deficient in GA biosynthesis. Therefore, the interactions between *Rht12* (gibberellin metabolism) with vernalization and photoperiod genes should be further investigated. Similarly, in barley, the photoperiod gene *Ppd-H1*, which is collinear with the wheat *Ppd* gene, was also associated with flowering time (Laurie et al., 1995). The dominant alleles at *Ppd-H1* confer early flowering under long days but have no effect under short days. In wheat, dominant *Ppd* alleles confer an early flowering phenotype in long day and short day conditions, resulting in yield benefits under certain environments (Cockram et al., 2007). The observation that Arabidopsis has similar floral responses to photoperiod and vernalization suggests that genes identified within its flowering pathways could play orthologous roles in temperate cereals. The interaction of the flowering control genes in wheat and barley with the circadian clock mechanism would also appear to be a fertile area for study in relation to crop improvement. In Arabidopsis, PRR genes have been implicated in providing adaptive responses to photoperiod in growth at different latitudes by modulating circadian timing (Michael et al., 2003). Plants in which the clock period is correctly matched to the day/night cycle are more photosynthetically efficient and productive than those grown in mismatched environments (Dodd et al., 2005).

The number of fertile florets is determined at the TS-AN phase, and hence this phase is particularly important for grain yield (Fischer, 1975; Flintham and Gale, 1983; Gonzalez et al., 2005; Rebetzke et al., 2012a). Lengthening the TS-AN duration in wheat would increase the number of fertile florets and finally improve yield potential (Slafer et al., 1990; Gonzalez et al., 2005). In this study, the *Ppd-D1b* plants (pp) achieved higher survival of florets and higher fertility than the *Ppd-D1a* plants (PP) in both dwarf and tall lines, probably due to the longer TS-AN duration (Gonzalez et al., 2005). However, the *Rht12* dwarf plants, especially the RRPP plants, which had the shortest TS-AN duration, achieved more fertile florets per spike than the tall plants, although the total number of florets initiated was less than that of the tall plants. This may be caused by the dwarf stature reducing the competition between the stem and spikes, and this could facilitate more dry matter transport to spikes during the TS-AN period, and in turn could result in producing more fertilized florets and grains per spike (Miralles et al., 1998; Rebetzke et al., 2012a; Chen et al., 2013).

It has been reported that *Ppd-D1a* typically advances flowering (Worland, 1996; Bentley et al., 2013; Wilhelm et al., 2013) and is associated with fewer spikelets per ear (Worland et al., 1994, 1998), reduced tillering (Worland et al., 1998), and reduced plant height by approximately 15.0% (Wilhelm et al., 2013). These effects were also observed in this study, and *Ppd-D1a* showed a significant reduction effect (9.7 cm, 10.2%) on plant height, though it was much weaker than that of *Rht12*. Similar to that reported before, plant height was reduced by 49.5 cm (43.2%) by *Rht12* in this study (Rebetzke et al., 2012a; Chen et al., 2013). However, *Ppd-D1a* has a similar reduction effect on plant height as the wheat dwarf gene *Rht8* (7–15%) (Tang et al., 2009; Rebetzke et al., 2012b; Wang et al., 2015). Actually, *Ppd-D1a* and *Rht8* are both located on wheat chromosome 2D and linked closely with each other (Worland, 1996; Pestsova et al., 2002; Gasperini et al., 2012; Chebotar et al., 2013). It was found that *Rht8* contributed a 13% reduction in plant height, while *Ppd-D1a* showed a 3% reduction through the analysis of recombinant inbred lines segregating at these two loci (Gasperini et al., 2012). The parent Jinmai47 in the current study contains both the dominant *Ppd-D1* allele and the dwarf allele of *Rht8*, assuming that tight linkage exists between *Rht8* and *Ppd-D1a* in the lines examined in this study, *Rht8* is likely the primary cause of the height reduction associated with *Ppd-D1a*. Moreover, it was also reported that *Rht8* is responsible for photoperiod insensitivity (Worland, 1996), and genotypes containing *Rht8* were early-maturing (Li et al., 2009), which may be correlated with *Ppd-D1a* due to their linkage. Because of the reduced plant stature, the *Rht12* dwarf plants (RR), either with *Ppd-D1a* or *Ppd-D1b*, had greater lodging resistance than that of the tall plants (rr), while the tall plants with *Ppd-D1a* (rrPP) had better resistance to lodging than rrpp plants.

The pleiotropic effects of *Rht12* on yield-related traits were evaluated in previous studies (Rebetzke et al., 2012a; Chen et al., 2013). Similar results were found in this study that *Rht12* increased the numbers of grains per spike and fertile ears per plant and the harvest index, while decreasing the grain size in both the PP and pp genotypes. It was reported that the reduced grain size was not the primary effect of *Rht* genes, but probably the results of the competition between the increased ear fertility and grain development under limiting photosynthate availability (Flintham and Gale, 1983). Furthermore, grain size can also be affected by flowering time, because the duration of the grain-filling stage under favorable conditions prior to harvest was shortened by the delayed flowering (Worland et al., 1994). However, *Ppd-D1*a significantly increased the thousand kernel weight, which may be a subsequence of the decreased number of fertile florets and the longer grain-filling duration of the PP genotypes.

In this study, the earlier flowering of 9–10 days in the PP lines may have benefited grain filling. However, RRPP plants achieved larger thousand kernel weight than RRpp plants, but this increased thousand kernel weight was still shorter than that of the tall plants (rr), indicating that the reduced thousand kernel weight of the *Rht12* dwarf lines may be affected by many other factors, such as the smaller size of flag leaf, peduncle, as well as other vegetative organs that probably could not provide enough assimilates available to produce large grains. Thus, for improving the thousand kernel weight of *Rht12* dwarf plants, genes involved in grain development should be introduced in the breeding program. Finally, the plant yields of the RRPP, rrPP, and rrpp lines were similar, while RRpp lines achieved the lowest plant yield. However, the RRPP genotype had a larger harvest index than the other three groups due to the higher plant yield and the smaller plant biomass. The effects of *Ppd-D1a* found in this study were similar to those reported previously (Worland et al., 1998; Foulkes et al., 2004; Guo et al., 2010; Cho et al., 2015; Chen et al., 2018). However, it is clear that the combination of *Rht12* and *Ppd-D1a* has advantages on obtaining a larger grain yield and a higher harvest index, which should be considered in the application of other dwarfing genes in wheat breeding. Moreover, based on the 3-year experiment, two lines named entry 8 and entry 10 in the RRPP category, respectively, were selected for further breeding tests. Entry 8 was the highest yield (15.3 g) line with the third lowest height (59.1 cm) on average. Entry 10 was the second highest yield line with 15.1 g on average and 60.7 cm in height with the most grain number per spike. These two lines were into the advanced selection trials for further tests.

## Conclusion

The *Ppd-D1a* allele could rescue the delaying effect of *Rht12* on spike development, and the *Rht12* dwarf lines when *Ppd-D1a* flowered earlier due to the shortened reproductive phase. This could promote the use of *Rht12* in wheat improvement. Moreover, the combination of RR and PP had no negative effect on plant yield, and some dwarf lines with good agronomic traits were selected for further use in dwarf and high-yield wheat breeding.

Future prospects: Flowering time is a complex trait in wheat and is influenced by a few major genes controlling vernalization requirement, photoperiod response, intrinsic earliness, as well as numerous small-effect QTL, which facilitate fine-tuning of flowering to different climatic conditions (Hanocq et al., 2004; Langer et al., 2014). *Ppd-D1* was found to account for almost half of the genotypic variance in flowering in some genetic background (Langer et al., 2014; Guedira et al., 2016; Würschum et al., 2018). It was also observed in this study that *Ppd-D1* was the major factor affecting flowering time in both the tall and dwarf plants. However, only one cross (Jinmai47 × Karcagi) was used in this study, and hence the effects of the different genes associated with flowering and yield from the two parents might not have been rigorously assessed. Therefore, more work is needed to explain the phenotype observed, such as using different genetic backgrounds and conducting experiments in different environments or in combination with other flowering-associated genes to achieve a comprehensive understanding of the interactive effects between *Ppd-D1* and *Rht12* on spike development, plant height, and other agronomic traits. Actually, new work is in progress in our group to obtain a better understanding of the effects of *Ppd-D1* in *Rht12*, *Rht5*, and *Rht14* dwarf plants and to breed earlier flowering dwarf germplasms (Chen et al., 2018).

## Author Contributions

LC and YD carried out the experiments. LC, YD, HC, CC, QL, and RM analyzed the data. JL, YY, LL, YC, XQ, HL, and SL assisted in the experiments. Y-GH and LC conceived and designed the experiments and wrote the manuscript. KM, HC, and FY helped revise the manuscript. All authors read and approved the final manuscript.

## Conflict of Interest Statement

The authors declare that the research was conducted in the absence of any commercial or financial relationships that could be construed as a potential conflict of interest.
