# Supplementary material for: The Photoperiod-Insensitive Allele Ppd-D1a Promotes Earlier Flowering in Rht12 Dwarf Plants of Bread Wheat
Source: Front Plant Sci. 2018 Oct 22;9:1312. doi: 10.3389/fpls.2018.01312 (PMC6204387; doi:10.3389/fpls.2018.01312)
Supplement: Supplementary file 1 [file Table_1.DOCX]

**Supplemental Table 1**. Sequence information of the markers used for detecting vernalization genes, photoperiod genes and dwarf genes.

| Locus | Allele | Primer | Sequence (5'-3') | Expected  band size  (bp) | Reference |
| --- | --- | --- | --- | --- | --- |
| *Vrn-A1* | *Vrn-A1a* | VRN1AF | GAAAGGAAAAATTCTGCTCG | 965,876 | Yan et al., 2004 |
|  | *Vrn-A1b* |  |  | 714 |  |
|  | *vrn-A1* | VRN-INT1R | TGCACCTTCCCCCGCCCCAT | 734 |  |
|  | *Vrn-A1c* |  |  | 734 |  |
|  | *Vrn-A1c* | Intr1/A/F2 | AGCCTCCACGGTTTGAAAGTAA | 1170 | Fu et al., 2005 |
|  |  | Intr1/A/R3 | AAGTAAGACAACACGAATGTGAGA |  |  |
|  | *vrn-A1* | Intr1/C/F | GCACTCCTAACCCACTAACC | 1068 |  |
|  |  | Intr1/AB/R | TCATCCATCATCAAGGCAAA |  |  |
| *Vrn-B1* | *Vrn-B1* | Intr1/B/F | CAAGTGGAACGGTTAGGACA | 709 | Fu et al., 2005 |
|  |  | Intr1/B/R3 | CTCATGCCAAAAATTGAAGATGA |  |  |
|  | *vrn-B1* | Intr1/B/F | CAAGTGGAACGGTTAGGACA | 1149 |  |
|  |  | Intr1/B/R4 | CAAATGAAAAGGAATGAGAGCA |  |  |
| *Vrn-D1* | *Vrn-D1* | Intr1/D/F | GTTGTCTGCCTCATCAAATCC | 1671 | Fu et al., 2005 |
|  |  | Intr11/D/R4 | AAATGAAAAGGAACGAGAGCG |  |  |
|  | *vrn-D1* | Intr1/D/F | GTTGTCTGCCTCATCAAATCC | 997 |  |
|  |  | Intr1/D/R3 | GGTCACTGGTGGTCTGTGC |  |  |
| *Ppd-D1* | *Ppd-D1a* | 2D-Ins-F1 | ACGCCTCCCACTACACTG | 288 | Beales et al., 2007 |
|  |  | 2D-Ins-R1 | GTTGGTTCAAACAGAGAGC |  |  |
|  | *Ppd-D1b* | 2D-Ins-F1 | ACGCCTCCCACTACACTG | 414 |  |
|  |  | 2D-Ins-R2 | CACTGGTGGTAGCTGAGATT |  |  |
| *Rht-B1* | *Rht-B1b* | BF | GGTAGGGAGGCGAGAGGCGAG | 273 | Ellis et al., 2002 |
|  |  | MR1 | CATCCCCATGGCCATCTCGAGCTA |  |  |
|  | *Rht-B1a* | BF | GGTAGGGAGGCGAGAGGCGAG | 273 |  |
|  |  | WR1 | CATCCCCATGGCCATCTCGAGCTG |  |  |
| *Rht-D1* | *Rht-D1b* | DF | CGCGCAATTATTGGCCAGAGATAG | 254 | Ellis et al., 2002 |
|  |  | MR2 | CCCCATGGCCATCTCGAGCTGCTA |  |  |
|  | *Rht-D1a* | DF2  WR2 | GGCAAGCAAAAGCTTCGCG | 264 |  |
|  |  |  | GGCCATCTCGAGCTGCAC |  |  |
| *Rht4* | *Rht4* | WMC317 | TGCTAGCAATGCTCCGGGTAAC | 170 | Ellis et al., 2005 |
|  | *rht4* |  | TCACGAAACCTTTTCCTCCTCC | 150 |  |
| *Rht5* | *Rht5* | BARC102 | GGAGAGGACCTGCTAAAATCGAAGACA | 200 | Ellis et al., 2005 |
|  | *rht5* |  | GCGTTTACGGATCAGTGTTGGAGA | 165 |  |
| *Rht12* | *Rht12* | WMS291 | CATCCCTACGCCACTCTGC | 250 | Korzun et al., 1997 |
|  | *rht12* |  | AATGGTATCTATTCCGACCCG | 300 |  |
| *Rht13* | *Rht13* | WMS577 | ATGGCATAATTTGGTGAAATTG | 130 | Ellis et al., 2005 |
|  | *rht13* |  | TGTTTCAAGCCCAACTTCTATT | 120 |  |
